# The lambs of sheepfoot lane: Oldham Athletic AFC 1960–2023; football, politics and identity

**DOI:** 10.3389/fspor.2024.1319963

**Published:** 2024-07-17

**Authors:** James O'Brien

**Affiliations:** ^1^ Independent Researcher, Switzerland; ^2^ University of Vic - Central University of Catalonia, Barcelona, Spain; ^3^ Birkbeck, University of London, London, United Kingdom

**Keywords:** Oldham Athletic, Boundary Park, Jimmy Frizzell, Joe Royle, globalisation, Oldham, politics, cultural identity

## Abstract

The article focuses on an overview of the history, politics and cultural identity of Oldham Athletic AFC between 1960 and 2023. During this period the club, a founding member of the Football League, played across six divisions of the game's pyramid. My hometown club, the Latics faced an existential threat to its survival following relegation to the National League in 2022, before being rescued and brought back into local ownership on the long road back to its recapturing its former status. The successes of the Frizzell and Royle eras (1969–1994) contrast with the decline of the club in the 1960s, and its gradual meltdown since the mid—1990s. These are interwoven with the economic, geopolitical and cultural dynamics of the game. The study locates the history of a small club geographically positioned within the vortex of the of the two Manchester giants and Liverpool, in English football's traditional hinterland of the industrial north west. The article has four main themes. Firstly, it examines Oldham Athletic as custodian of local identity, fusing folkloric rituals within the club's historic and contemporary nexus. Secondly, it contextualises the interface between global, glocal and grobal forces impacting on tradition, modernity and post- modernity in the club's lexicon. It then considers the themes of ownership, commodification and globalisation, debating whether Oldham Athletic stands as a bastion of anti- globalisation, or reconfirms existing global patterns at the micro level, incorporating salient theoretical perspectives in this analysis. Finally, the review evaluates whether the political and social class cleavages of the club's roots in Oldham's late Victorian industrial landscape still resonate, or if they have been marginalised by socio- economic changes since 1960.

## Introduction

The tenure of Ken Bates as chairman of Oldham Athletic from 1965 to 1968 was seldom far from the press headlines. Ambitious, brash and burnishing cash to match his enormous ego, he arrived in a blaze of publicity to save the struggling third division club, promising “Europe by 1970”, only to controversially resign, a year before the Latics returned to the basement division of the football league in 1969, precisely where they had started a rollercoaster decade in the club's history (1). Bates coined the term “The Lambs of Sheepfoot Lane” in an angry tirade against the club's fans, thinly masking his prejudices against what he saw as the narrow working- class beliefs of the Latics' core supporters (2). This exacerbated the split between chairman and fans. His exit was ignominious, leaving the club on the edge of a precipice (3, 4). As Bates departed, so Jim Williams, the doyen of football reporters, entered its folklore and history, covering every aspect of the Latics with independence and distinction for the “Oldham Evening Chronicle” until the late 1980s. Bates' caricature of the club survived, transferred to the performances of the team in the 1960s, when strong home form contrasted with poor results away from Boundary Park (4). Through the pen of Jim Williams, I read about the horror 5-0 defeat for Jimmy McIlroy's side at Torquay United in 1966, the apocalyptic 8-1 humiliation at Peterborough United in the relegation season of 1968–69, and the 5-0 meltdown at Bournemouth in January 1971, a rare stain on a promotion season (1, 3).

This review is part of ongoing research into the history, politics and cultural identity of Oldham Athletic AFC: I gave a paper on the subject at the Sport and Politics Study Group Conference hosted by the University of Aveiro in June 2022. It contextualises the themes forming the basis of a proposed single author study of the club, to be published in 2026. It has two main methodological approaches. It considers the secondary literature about the club, whilst also examining studies of the history, politics and culture of Oldham itself. This approach is matched by witness accounts derived from more than fifty years supporting the team since August 1966 (5). This fan's journey has encompassed many highs and lows. I was there in the “Chaddy End” in December 1974, watching the Latics beat Manchester United 1-0 in a league match on a quagmire of a pitch; Sixteen years later, I witnessed a pulsating 3-3 draw at Maine Road between the same opponents, when the plucky Latics took their illustrious opponents all the way (6, 7). I saw an abysmal 2-1 home defeat to non—league South Shields in a first round FA Cup replay, shivering in the Rochdale Road end in a sparse huddle of shellshocked fans; I watched with unbelieving eyes the 6-0 humiliation by Aston Villa, captured by Granada Television in 1971, a dismal low- point in the club's return to the Third Division (2, 4).

The review provides an insight into the world of “Oldham, Brave Oldham”, a metaphor for the town characterising the club and its teams down the ages: the spirited underdog, frequently punching above its weight, giving many a goliath a run for their money from the frozen, windswept terrain of Boundary Park on icy winter nights, one of the coldest places on earth, nestling in the bleak, austere foothills of the Pennines (8). In many ways Oldham Athletic represents the good days and hard times of Oldham itself, an occasional escape from harsh economic realities, as in the early 1970s, when the team's success contrasted sharply with industrial decline and political conflict (1, 9, 10). The team and the town live cheek by jowl, hand in glove, a keen sense of localism striking out against the mega—metropolis of Manchester (11).

## Oldham athletic: key themes and issues

Oldham Athletic grew out of the demographic and socio—economic changes in Oldham during the second half of the nineteenth century. The club's foundation as Pine Villa in 1895, before it was renamed in 1899, coincided with the town's population growth from 52, 820 in 1854 to 137.000 in 1901, fuelled by its expansion at the heart of the textiles industry in late Victorian and Edwardian England (3, 9, 10). This had manifold implications for Oldham, and continued the rise of the new bourgeoisie, the self- made men of the industrial revolution. The parallel growth of sport, in particular the foundation of the professional English Football League (1888), fostered the urban milieu in which clubs such as Oldham Athletic could locate roots deep within its burgeoning community (12, 13). This bond between the club's fortunes, and those of the town has created a close, though not uncritical relationship between players, fans and team. This has led to suggestions that the club is trapped in a time warp, stuck in its industrial and post-industrial past, with a rich history but stubbornly refusing to embrace the changes of the contemporary game (14, 15). Although the red of United and the blue of City pushes them into an enclave Latics fans are loyal, outspoken and resilient, proud of their town and their club, with a sharp sense of humour (1, 16). The Clayton Green pub, next to the “Chaddy End”, was the forum for many a post—match discussion, when players came in to receive praise and admonishment. These rituals confirmed the survival of the folkloric grassroots of local identity within the football—community axis (12, 15).

The relationship between football and the many facets of globalisation is a seminal arena of debate in the contemporary game (17–19), The success of Oldham Athletic in the late 1980s and early 1990s, culminating in the ‘Pinch Me! ‘ season of 1989–1990, when the club challenged for honours on three fronts, came at a time when English football was undergoing seismic changes, leading to the foundation of the Premier League in 1992 (17, 20). The heroic deeds of the Latics playing outstanding football captured the imagination of the viewing public, and was soon followed by promotion to the First Division in 1990–1991, returning the club to the top flight for the first time since the 1920s (7, 14). Humble Oldham Athletic was embroiled in the midst of the power shift from the dominant forces of tradition and modernity to the post- modern globalism of media rights, product branding and corporate capitalism as stars became celebrities and fans became consumers (17). The club had climbed a mountain to reach the pinnacle of the English game; in the early 1990s it stood at the crossroads of either trying to become part of the globalisation process of commodification, gentrification and consumerism, bucking the trend to compete with the elite, or to be the antithesis to globalisation, stressing tradition, localism and grassroots identity (7, 16). As the 1990s unfolded, the Latics, like Icarus having flown too close to the sun, entered a period of gradual decline, reverting to being a small club surrounded by footballing giants (2). The club provides an insight into the relationship between football and competing theories of globalisation, encompassing economic, geopolitical and cultural considerations (19, 20). The analysis afforded by the mass consumerism of the Mc Donaldization of Society critique elucidates an invaluable backdrop as to how clubs like Oldham Athletic reflect this model (21).

The stereotypical construction of Latics fandom is local, white working -class male, replete with cloth cap and a pie and pint at half-time, cast immortally in the mould of the team from a town of chimneys (1, 10). Since 1945, the town's two parliamentary constituencies Oldham East and Saddleworth and Oldham West and Royton (in which Oldham Athletic is situated), have been mostly held by the Labour Party, embracing changes in electoral boundaries (9, 10, 22), tending to reinforce the stereotype. However, there have always been caveats within these broad assumptions. Working—class Conservatism and support for the Liberals, especially in more affluent Saddleworth (22), the rise of the National Front, British National Party and UKIP, together with falling turnout and voter alienation, have all suggested political volatility at different periods since 1960 (9, 10). The detailed study emanating from this review will consider the Latics- Labour- Oldham matrix as more research is needed on this fluid mosaic, at parliamentary and local levels. In a similar vein, the club's fandom has historically been more fluid than the cloth cap image suggests, embracing the aspirational middle class of Saddleworth, local businesses and the patronage of wealthy local supporters (3, 6). It has evolved during the period of this overview, with the increase in female fandom and a wider cross section of the town's declining population (7).

Finally, the history of the club, its place in the globalisation debate, and its links to local and national politics should be considered in respect of its articulation of social class. The synthesis between English football and social class is symbiotic and organic (13). Clubs such as Oldham Athletic are examples of the class stratification model, in that its history, tradition and culture are locked into the critical political, demographic and socio—economic changes that took place in the second half of the nineteenth century. Within this nexus the working- class appropriation of the sport found its most potent expression in the industrial heartland of north- west England (12). The Latics were one of a cluster of clubs which grew out of this landscape, each carving their distinctive niche in the game's development. This engendered a narrow, class centred ideology around football, which shackled it in a cocoon of entrenched values and attitudes, making change slow and tortuous. The team from the mill town, deep in industrial Lancashire, neatly matches this hypothesis (1, 12). This differs from the class mobility approach, which considers the capacity of sport to break down barriers of social class, creating more meritocratic scenarios (17, 23). In the 1960s football was locked into traditional hierarchies of class orthodoxy (24); Demographic change, the growth of gender politics and the post- industrial landscape all impacted on football's representation of social class. From the mid -1970s the roots of the Latics' folkloric identity were shaken as the club struggled to adapt to a changing society and issues of inclusion and exclusion (1, 9, 17).

## Discussions

In 2023, following years of decline, which saw the club's performances, image and reputation take a battering as it became the butt of gallows humour, Oldham Athletic looks ahead with cautious optimism. Whilst there may never be a return to the glory days of the Royle era, there is some sense that the club, its new ownership fashioned from the local support and patronage of lifelong Latics fan Frank Rothwell and his family, has survived an apocalyptic demise and is on the road to recovery. This raises the question as to where the club's ambitions lie, beyond the stated objective of a return to the Football League. The Premier League is unimaginable, such has the game's geopolitics changed since the Latics played in it during season 1993–1994. The glass ceiling is now a solid, efficiently run mid- table club in the EFL Championship; well supported by loyal fans, with the odd cup upset to evoke nostalgia, boost income and give the team and town media profile. Even this scenario seems a distant dream given the current status of the club. The “ghost town” notion of the Latics becoming a living museum in the hinterland of the football pyramid strikes uncertainty in keeping the club's identity alive in that post—industrial space where clubs such as Bury, Southport and Chester have been diminished or almost vanished (12, 17).

Between 1960 and 2023 the Latics embraced the global, the glocal and the grobal as the club moved up and down six different divisions: The early 1990s witnessed flirting with the global until that ambition was extinguished by Mark Hughes' goal in the last minute of extra time in the 1994 FA Cup Semi-Final at Wembley, denying the club the realisation of Ken Bates' European promise from the 1960s: Many fans believe that this fortuitous goal struck a blow at club's heart from which it never recovered (7). The club has danced with permutations of the glocal since the 1970s, when the Frizzell era saw a renaissance of local pride in the team's success as it moved up the leagues, fusing localism and ambition in football's enhanced media and international profile (1, 3). During the 1980s and 1990s, before decline and realism set in, Oldham Athletic toyed with the grobal; the notion that the team's success could be a catalyst for the regeneration of post- industrial Oldham. This was a time when the club gave the town hope against the ravages of Thatcherism and mass unemployment (10). These hopes proved to be a temporary respite from the impacts of long-term decline. In 2023 the Latics could be an irrelevant sideshow in this debate. In this matrix localism and community are at the forefront of future discussions.

The history and identity of Oldham Athletic is bound up in modernity (23). The review covers the club's contemporary history as football transitioned unevenly into post- modernity (17). By the 1980s this constituted an identity crisis for the Latics, the ephemeral on field successes of the period masking seminal changes brought about by the game's burgeoning, mediatisation and globalism (17, 18). The evidence suggests that the club has been unable to deal with the impacts of these impulses, which ultimately pushed the Latics to the brink of extinction in the post-pandemic world of 2022. The future of the club, in protecting, consolidating and promoting its identity is tied up in how it responds to the changing dynamics of the contemporary game.

Reflecting on the period 1960–2023 in the club's history, what is the club's legacy, its imprint on the town, through all the false dawns and multiple struggles, on and off the pitch? What will inspire the next generations of fans to ignore the pull of City and United and support their hometown team? The answer lies in the achievements of managers and players. Three managers and five players characterise the decades covered by this review. From 1969 until 1994, the Latics had only two managers: This contrasts with the plethora of coaches who occupied the manager's desk when the club was in freefall from 2017 to 2022. Jimmy Frizell and Joe Royle were benchmarks for progress and stability: between them they took the Latics from the foot of the then Fourth Division to being founder members of the Premier League (3, 7, 16). Frizzell, the Saviour, served the club for twenty-one years as player, coach and manager (1, 2, 4). The Latics faced the prospect of seeking re-election when he took over in December 1969; within five years, on the back of two promotions, the club had returned to the second division for the first time since the 1950s (3). Frizzell's replacement was the inexperienced Joe Royle (16). Royle, “The Juggler” scaled the heights with the Latics, before being tempted by Everton, his first club. In his twelve years at Boundary Park, he strode the club like a colossus, bringing many wonderful moments and reawakening the bond between the team and the town. A third manager completes this trinity: “Houdini” John Sheridan; a stylish midfield player with Leeds and Sheffield Wednesday, he served the Latics as player, coach and manager on several occasions between 2006 and 2022, saving the club from relegation to League Two, putting a temporary halt to the decline of the club. Sheridan had the Latics in his heart, and came closer than anyone in bringing the good times back to Boundary Park.

As with these celebrated managers, the legacy of the club lives in the deeds of those players whose names were chanted by the “Chaddy End”. Five stand out from the journeymen, starlets, old stagers, entertainers and rejects I saw grace the muddy turf and plastic paradise of Boundary Park. Big, burly Jim Fryatt, with his fearsome sideburns, was one of Frizzell's inspired early signings; the partnership he forged with the lightning -fast David Shaw terrorised Fourth Division defences during the promotion season of 1970–71, their goals a vital ingredient of a free-scoring side (1). The talent of Alan Groves lit up the Third Division championship campaign in 1973–74: his speed and wing play made him a fans' favourite before his premature death in 1977 (3). As the Latics settled into the Second Division in the late 1970s, the homegrown talent of Graham Bell was the creative midfield heartbeat of the team, following in his father's footsteps in playing for the club.

Royle brought many wonderful players to Oldham Athletic. Two “rejects” were key components of the late 1980s, early 1990s team. Andy Richie had burst onto the scene as a teenager at Manchester United, before his career stalled at Brighton and Leeds. Royle brought him to the Latics in 1987; he went on to star in the following years, a scorer of spectacular goals against the best teams in the country (7). For much of his time at the Latics, Richie was supported by a Manchester City's reject, the silky skills of the “Black Flash” Roger Palmer proving a perfect foil to harness the attacking instincts of Royle's swashbuckling side (16). These players epitomised the Latics in the two most successful periods of the club's recent history, keeping its identity alive and ensuring that the “Lambs of Sheepfoot Lane” carved their own chapter in English football.
